# “Bedside-to-Bench” Behavioral Outcomes in Animal Models of Pain: Beyond the Evaluation of Reflexes

**DOI:** 10.2174/1570159X113119990041

**Published:** 2013-12

**Authors:** Enrique J Cobos, Enrique Portillo-Salido

**Affiliations:** 1 Department of Pharmacology, School of Medicine, University of Granada, Avenida de Madrid 11, 18012 Granada;; 2 Drug Discovery and Preclinical Development, Esteve, Avenida Mare de Déu de Montserrat 221, 08041 Barcelona, Spain

**Keywords:** Analgesia, animal model, anxiety and depression, behavioral outcomes, functional disability, pain, sleep, spontaneous and motivated behavior.

## Abstract

Despite the myriad promising new targets and candidate analgesics recently identified in preclinical pain studies, little translation to novel pain medications has been generated. The pain phenotype in humans involves complex behavioral alterations, including changes in daily living activities and psychological disturbances. These behavioral changes are not reflected by the outcome measures traditionally used in rodents for preclinical pain testing, which are based on reflexes evoked by sensory stimuli of different types (mechanical, thermal or chemical). These measures do not evaluate the impact of the pain experience on the global behavior or disability of the animals, and therefore only consider a limited aspect of the pain phenotype. The development of relevant new outcomes indicative of pain to increase the validity of animal models of pain has been increasingly pursued over the past few years. The aim has been to translate “bedside-to-bench” outcomes from the human pain phenotype to rodents, in order to complement traditional pain outcomes by providing a closer and more realistic measure of clinical pain in rodents. This review summarizes and discusses the most important nonstandard outcomes for pain assessment in preclinical studies. The advantages and drawbacks of these techniques are considered, and their potential impact on the validation of potential analgesics is evaluated.

## 1 CHALLENGES OF PRECLINICAL PAIN RESEARCH: HISTORICAL PERSPECTIVE OF THE DEVELOPMENT OF CLINICALLY RELEVANT PAIN MODELS AND THE STANDARD CONCEPTUALIZATION OF NOCICEPTION

Millions of people world-wide suffer from chronic pain [1]. Pain management is an unmet clinical need and the development of new analgesic drugs with better efficacy/tolerability than those currently available is a high priority [2]. The final aim of pain research is to translate the basic scientific data into clinically useful new analgesics. However, in spite of increasing efforts by basic pain researchers, little translational progress from “bench-to-bedside” has been achieved [3], and most of the “new” pain medications consist of refined delivery methods for known analgesic compounds, or combinations of known analgesics (the so-called combination drug therapy), with almost no addition of truly new analgesics targeting novel mechanisms [4-6]. The development of new analgesic drugs depends strongly on the predictive validity of preclinical animal models [7], and therefore there is a need to refine preclinical pain behavioral testing of candidate analgesics [6,8-12]. The main challenges of preclinical pain research are twofold: the development of animal models (understood as the experimentally induced injury) to closely resemble relevant human pain conditions, and the search for adequate behavioral outcomes to properly evaluate the pain experienced by the rodents, and hence the analgesia induced by successful treatments.

From the early times of preclinical pain research, when studies were devoted to acute nociception (mainly using thermal stimulation in the hot plate and tail-flick tests), several advances have been obtained in the search for pain models relevant to human pain conditions. Historically, one important advance in animal pain models was achieved with the development of models of more sustained (tonic) pain, including the intraplantar injection of formalin (formalin test) and the intraperitoneal injection of chemical algogens such as diluted acetic acid or phenylbenzoquinone (writhing test). The pharmacological sensitivity of acute and tonic pain models was astonishingly different, and both opioids and nonsteroidal antiinflammatory drugs (NSAIDs) needed much lower doses to ameliorate pain responses in tonic pain models than in acute pain. This was particularly important, since the doses of analgesics needed to induce anti-nociceptive effects in tonic pain models were closer to the doses used for clinical pain in humans than the very high doses of analgesics effective in models of acute thermal nociception in rodents (reviewed in [13]). This differential sensitivity to drug-induced analgesia played an important role in the shift in the conceptualization of the type of pain being explored in preclinical pain research, and inspired researchers world-wide to develop the current more clinically relevant models of pain in rodents. Some of these animal models attempt to mimic inflammatory, osteoarthritis, neuropathic, cancer and postoperative pain in rodents. Inflammation can be induced experimentally by several agents, including carrageenan, complete Freund’s adjuvant (CFA) or bacterial lipopolysaccharide (LPS) [13-15]. Other inflammatory pain models involve inflammation of the viscera, such as cystitis induced by cyclophosphamide (e.g. [16]). Osteoarthritis can be induced experimentally by several methods, including the injection of the chondrocyte glycolytic inhibitor monoiodoacetate (MIA) or by surgical destabilization of the medial meniscus [17]. The most widely used models of traumatic nerve injury include chronic constriction injury (CCI), constriction of the saphenous nerve (CCS), partial sciatic nerve ligation (PSNL), L5/L6 spinal nerve ligation (SNL), tibial nerve transection (TNT), L5 spinal nerve transection (SNT) and spared nerve injury (SNI) [18,19]. Peripheral neuropathy can also be induced experimentally (and clinically) by antineoplastics, including paclitaxel, vincristin, cisplatin or oxaliplatin [18,20,21]. In addition to peripheral neuropathic pain models, neuropathy can also be induced by damage to the central nervous system by mechanical contusion or electrolytic lesions, among others [18,22]. Cancer pain is usually induced in rodents by injecting tumor cells into the bone marrow (femur, calcaneus or tibia), which is accompanied by bone destruction [18]. To model postoperative pain, the first proposed model was plantar incision [23] and more recently, more realistic surgical procedures have been used, such as laparotomy [24,25] or thoracotomy [26]. These different injuries attempt to model clinical pain states in order to study their pathophysiological mechanisms and test new candidate analgesics. These animal models undoubtedly more closely mimic relevant human pain conditions than the evaluation of acute pain.

If translating pain conditions from humans to rodents is not devoid of challenges, finding pain outcomes in rodents equivalent to those seen in human patients is no bed of roses either. From the very beginning of preclinical pain research, studies focused on the analysis of specific pain outcomes occurring in response to sensory stimulations of different nature. Initially, in studies of acute nociception, pain was mainly assessed as the latency to the appearance of reflexive movements of the stimulated area (the paw or tail for the hot plate or tail-flick tests, respectively). Similarly, in tonic pain models, either formalin injected into the hindpaw or the intraperitoneal injection of chemical algogens were also shown to be able to elicit pain-induced reflexive responses (flinching/licking of the affected paw or twitching of the body, for the formalin and the writhing tests, respectively) (reviewed in [8,13]). Later studies of pathological pain models focused on evaluating the enhancement of pain-like responses evoked by either mechanical (von Frey and Randall-Selitto tests) or thermal stimuli (Hargreaves and acetone drop tests) applied acutely. The von Frey test uses calibrated plastic hairs of different diameters (intensities of stimulation) which are applied sequentially to the target area of the animal to estimate the mechanical threshold for reflex withdrawal [27]. The Randall-Selitto test, originally developed in 1957 [28], consists of the application of increasing pressure *via *blunt mechanical stimulation to the hindpaw until a withdrawal reflex, struggling or vocalization appears (reviewed in [13,29]). Recent modifications of this test use fixed pressure to the paw and record the latency to the first pain-like response [30,31]. The Hargreaves test uses a controlled light beam directed to the paw from the bottom of a glass surface where the animal is placed, and the outcome measure is latency to the paw withdrawal reflex [32]. Finally, the acetone drop test is based on the sensation of cold produced by the evaporation of acetone placed on the paw, which might elicit pain-like responses under pathological conditions [33]. The outcome measures vary from the number or frequency of brisk foot withdrawals to the time spent reacting by licking or shaking the stimulated paw (reviewed in [13,29]). Alterations in these outcomes illustrate the sensory hypersensitivity (hyperalgesia and allodynia) which often accompanies a chronic pain condition, and constitute the standard measure of pain in preclinical pain research laboratories around the world. Therefore, in spite of the sophistication of the injuries used to replicate clinical pain in rodents, the dependent measures varied little and currently rely mostly on the use of reflexive outcomes, as previously reported [8,10,11,34]. However, pain is a subjective experience which is far more complex than a reflex in response to sensory stimulation. Pain perception is an aversive experience with complex consequences (e.g. disability, anxiety, depression) which alters the daily living activities and quality of life of human patients [1,2,35]. Therefore, evaluating pain in animals with exclusively reflexive outcomes is an over-reduction of the pain experience, and this simplification of the preclinical evaluation of pain may have a negative impact on the bench-to-bedside translation of potential clinically useful compounds [6,8-12]. A number of recent animal studies, summarized in the following sections, aimed to study pain based on its consequences rather than assessing specific behavioral pain responses. In this sense, every behavioral change attributable to pain during a painful condition can potentially be considered a pain behavior and therefore useful to assess both pain and the efficacy of analgesic treatments. These outcomes aim to provide a more realistic view of the influence of pain on the activities of rodents as a way to evaluate the human “everyday pain experience” more accurately, and include the monitoring of physical disability, spontaneously emitted behaviors, aversion to pain, the rewarding properties of analgesia, and psychological alterations during a pain condition. We will analyze and highlight both the advantages and drawbacks of these approaches, their possible usefulness for testing candidate analgesics, and as possible points for improvement.

## 2 MEASURING FUNCTIONAL DISABILITY IN RODENTS: ADAPTIVE POSTURAL CHANGES INDUCED BY PAIN AND GRIP STRENGTH DEFICITS

In human patients, a painful condition might induce postural adaptations and can limit motion, thus contributing to physical disability. Distorted posture during resting or ambulation have long been monitored in clinical research as pain indicators (e.g. [36-41]). A different indicator of motor disability used in human patients is the decrease in grip strength, which has been shown to be associated with daily pain in a variety of painful pathologies, including musculoskeletal disorders such as osteoarthritis and rheumatoid arthritis (e.g. [42-48]). As putative pain indicators the consequences of a painful condition on postural changes in rodents as well as grip strength deficits have been assessed quantitatively.

### 2.1 Adaptive Postural Changes Induced by Pain: Weight Bearing Asymmetry and Gait Alterations

The weight bearing distribution in humans or rodents is normally approximately equal in each lower extremity. However, in conditions of unilateral hindlimb injury, body weight support is shifted to the noninjured side. Weight bearing asymmetry has long been targeted in preclinical pain research. Initially, this measure was obtained by heavily restraining the animals with a jacket and measuring the pressure exerted by each hindlimb with a transducer [49]. Later studies used the opposite approach, in which animals were forced to walk against the motion of an elevated rotating cylinder, and the time spent avoiding physical contact of the injured limb with the cylinder was automatically recorded [50]. It was in 1994 when Schott and coworkers developed the device in its present most popular form, in which the animal is not forced to perform any physical activity and is minimally restrained in a plexiglass chamber. The pressure exerted by each hindlimb is independently measured by force plates on the floor. Recently, new versions of this test have been developed in which rodents are allowed to move freely in a chamber designed to measure the pressure exerted by each limb in contact with the floor [51-54]. Changes in weight bearing distribution can be observed during hindlimb inflammation induced by the inflammatory agents most widely used in pain research (carrageenan, CFA or LPS) (e.g. [50,52,55-58]), in the experimental osteoarthritis model induced by the intraarticular administration of MIA (e.g. [59-61]), after paw incision [62] or during peripheral neuropathy [63,64].

Gait changes, assessed by walking tracks analysis, have been used in the past to study the functional motor consequences of neurological deficits in rodents [65-67]. Recently, complex devices have been designed for the in-depth study of gait in rodents (analyzing interlimb coordination, swing duration, stride length, paw print area, and in some cases weight bearing while walking) during free walking (CatWalk system) (e.g. [68]) or during forced locomotion (Digigait system) (e.g. [69]). These methodologies have been adapted very recently for measuring adaptive dynamic postural changes during a pain condition. There have been reported alterations in gait parameters during unilateral inflammatory insult [70,71] or peripheral nerve injury [63,71,72].

#### 2.1.1 Postural Changes During Inflammatory and Osteoarthritis Pain: Drug Effects and the Significance of the Outcome

Weight bearing asymmetry seen during hindlimb inflammation has been pharmacologically validated as a pain measure using standard analgesic treatments (e.g. [50,55,57,58]) (see Table **1**). Interestingly, weight bearing changes induced by inflammation were shown to be more sensitive to the ameliorative effect of NSAIDs than reflexive paw withdrawal (induced by von Frey stimulation or paw pressure), indicating a higher sensitivity to drug effects for nonreflexive than for standard reflexive outcomes [57]. Some potentially useful new pain targets have been tested in this outcome for inflammatory pain. The partial GABA_A_ receptor-positive modulator NS11394A greatly improves this outcome in rats with inflammatory pain, and at doses much lower than those needed to reverse mechanical allodynia [56]. As for the amelioration of weight bearing asymmetry during inflammatory pain, it has been shown that conventional analgesics can also reverse the effects of inflammation on gait [70,71], indicating that this outcome can be useful to test the analgesic-like activity of drugs on inflammatory pain.

Postural changes have also been used to test drug effects during MIA-induced osteoarthritis (in the early or the late phase). In general, NSAIDs have ameliorative effects on weight bearing asymmetry during the early phase after MIA injection, in which prominent inflammation develops, but limited efficacy in late stages of the disease [53,59,60,73,74] (Table **1**). This is in agreement with human studies showing that osteoarthritis pain is resistant to NSAIDs [75,76]. Two recent studies have shown analgesic-like effects of the pharmacological blockade of the potential new pain target TRPV1 using weight bearing asymmetry as the behavioral endpoint. Antagonism of this receptor ameliorates MIA-induced osteoarthritis in both the early and the late stages, although it was more effective in the former [61,77].

Assessing postural changes as a pain indicator is not free from motor confounders, since sedative drugs can also have an impact on weight bearing asymmetry. For example, diazepam ameliorates weight bearing differences during inflammation, but only at doses inducing obvious motor impairment [56]. These results suggest that drug-induced sedation and motor deficits might influence this outcome measure and result in false-positive analgesic-like effects.

In human patients, pain or anticipated pain play a role in guiding motor control. This is an adaptive mechanism aimed at protecting the body segment from the pain or the threat of pain, and reflects the fear-related aspects of pain (e.g. [78-80]). Although these postural changes in rodents have been suggested to be a measure of spontaneous [10,18] or evoked pain (by movement or by the pressure exerted by the contact of the injured limb with the floor) (e.g. [72]), they might be more consistent with the definition of a pain-avoidance behavior (i.e. fear of pain) [60,63,81], since by analogy with humans, they are probably the result of the avoidance of an activity which might induce pain or that aggravates existing pain. Regardless of the mechanistic nature of the behavioral changes observed and the exact procedure to measure them, the face validity of this outcome relies on the fact that it measures spontaneous postural changes equivalent to those seen in human patients during an asymmetrical injury (at rest or during movement), and the reversion of these changes by appropriate therapeutic intervention [37,38,40,41].

#### 2.1.2 Postural Changes During Peripheral Nerve Injury: Drug Effects and the Significance of the Outcome

In spite of the successful results in models of inflammatory pain or osteoarthritis, little success has been achieved in peripheral neuropathic pain models (Table **1**). Morphine, gabapentin, EMLA cream (2.5% lidocaine, 2.5% prilocaine) and the serotonin–norepinephrine reuptake inhibitor (SNRI) milnacipram, at doses effective against tactile allodynia measured with von Frey filaments, showed no or borderline effects on weight bearing differences during peripheral nerve injury [8,64,82]. In addition, the known antineuropathic drugs gabapentin and duloxetine, at doses able to reverse mechanical hypersensitivity, were unable to restore neuropathy-induced gait deficits [71]. The differential sensitivity to analgesic drugs of both weight bearing asymmetry and gait alterations in inflammatory/osteoarthritis pain and peripheral nerve injury suggest that pain is the primary driver of postural changes in models of inflammatory and osteoarthritis pain, but not in peripheral nerve injury. Therefore, in the latter, these outcomes probably reflect perturbation of the motor system due to surgical damage to the motor axons, rather than pain.

### 2.2 Pain-induced Grip Strength Deficits

When a rodent is held by the tail, its natural reaction is to try to grasp anything available. When a rodent is placed on a wire mesh grid connected to a force transducer and then pulled away from the grid, the force transducer records the peak force exerted by the animal before releasing its grasp on the wire mesh grid. Grip strength assessment in rodents was developed to assess muscle relaxation and the integrity of the neuromuscular system in pharmacological and toxicological studies [86-88]. This technique has been adapted recently for the study of pain resulting from deep tissue inflammation or from cancer [89-91], and has been used more recently to test MIA-induced osteoarthritis pain [77,92]. Decreased grip strength under these conditions was reversed by known analgesics (Table **2**). In addition, this outcome was successfully used recently to test putative new pain targets, including CB (cannabinoid receptor) agonism for inflammatory and cancer pain [89], and TRPV1 (transient receptor potential vanilloid 1) antagonism and ERK (extracellular signal-regulated kinase) inhibition for osteoarthritis pain [77,93] (Table **2**). One obvious characteristic of grip strength determination to test the analgesic activity of compounds is that substances producing significant sedative/hypomobility or muscle weakness will decrease the target behavior, as illustrated, for instance, by the decrease in grip strength induced by the antipsychotic haloperidol and high doses of morphine in pain-free animals [92].

Although grip strength is considered a quantitative measure of function, in human patients this outcome is known to be affected by both physical impairment and psychological factors. For instance, poor grip strength may be influenced by fear of maximal contraction because of the expected increase in pain [43,45,47,94]. Although it is unknown whether the same psychological factors apply to rodents in this context, this outcome can represent a more complex pain phenotype rather than a simple index of musculoskeletal function.

## 3 PAIN-INDUCED CHANGES IN SPONTANEOUS BEHAVIORS: POTENTIAL MEASURES OF WELL-BEING IN RODENTS

Some methods designed to evaluate general well-being in human patients suffering from a chronic pain condition go beyond the direct measure of physical functioning, and evaluate the impact of pain on daily activities. These methods include questionnaires such as the Western Ontario and McMaster Universities Arthritis Index (WOMAC). Other questionnaires use patient preferences to determine the impact of pain in their quality of life. These include the McMaster Toronto Arthritis patient preference questionnaire (MACTAR) and the patient-specific index (e.g. [95-98]).Therefore, pain in humans (and hence the efficacy of therapeutic interventions) can be indirectly assessed by changes in their activities. As described in detail below, several approaches have been used to study the impact of pain on spontaneous (not forced) activities in rodents, and the possible usefulness of these measures for testing drug-induced analgesia.

### 3.1 Interference by Pain in Daily Living Activities of Experimental Animals: Home Cage Locomotor Activity

Alterations in home cage locomotion during chronic inflammation or nerve injury have been explored in an attempt to detect global behavioral changes in the daily living activities of rodents during chronic pain. Although some authors anecdotally reported differences in rat home cage locomotor activity during unilateral chronic inflammation [99], others were unable to detect clear differences in either rats or mice [100,101] (Table **3**). One recent report studied home cage locomotion using standard models of unilateral peripheral neuropathy (CCI and SNI), and found a slight deficit in animals with CCI and no observable deficit in animals after SNI [101]. One possible explanation for the discrepant results found in the home cage studies is that locomotion might be influenced by housing conditions that are not frequently reported, such as the bedding used, environmental enrichment and handling of the rodents by personnel, among others. These factors can make the conditions of the laboratory unique and difficult to reproduce. In addition, it should be taken into account that rodent home cages in animal facilities are not a natural environment, and the possible impact of pain in their natural habitat is unknown for practical reasons. Another possibility is that the most commonly used pain models, in which a single paw is usually injured, are insufficiently severe to drive consistent changes in the behavior of the animals (although sufficient to induce sensory hypersensitivity in the affected area), and therefore do not mimic the key characteristics of chronic pain in humans. This was suggested in an early study showing that single limb inflammation does not change home cage locomotion, and that bilateral inflammation is needed to induce deficits in this behavior [100]. Further experiments using biotelemetry showed that bilateral CFA-induced inflammation was able to markedly decrease home cage locomotion, and that this deficit was sensitive to the NSAID indomethacin [102]. The same approach was used to study the impact of bilaterally MIA-induced osteoarthritis on home cage activity [103] (Table **3**). Although the deficits in home cage locomotion during bilateral injuries appear clear, the possible usefulness of this methodology for analgesic drug testing still needs to be established.

### 3.2 Interference by pain in spontaneous behaviors in rodents: innate and motivated behaviors

Obviously, the spontaneous and preferred activities of humans are not the same as for rodents, and behaviors with ethological validity should be used to measure the overall impact of pain on the activities of the rodents. For the purposes of preclinical drug testing, these behaviors should be performed spontaneously and at a rate high enough to be readily measured during relatively short observation periods [104]. Innate behaviors (such as exploratory locomotion and burrowing), and motivated physical activity (such as locomotion in running wheels) meet these requirements, since it has been known for decades that rodents robustly and spontaneously perform these behaviors, which have been widely used in the past for experimental purposes other than pain assessment (e.g. [105-107]).

#### 3.2.1 Interference by Pain in Spontaneous Innate Behaviors: Exploratory Locomotion and Burrowing

The interference by pain in innate behaviors and the effects of analgesics have been explored. These behaviors include exploratory locomotion and burrowing behavior. Exploratory locomotion is driven by factors different from home cage locomotion. The driving force of exploratory locomotion is the novelty of the environment, which boosts basal activity and increases the window for the reliable detection of suppression by pain. Decreases in exploratory behavior are consistently reported during hindlimb inflammation (e.g. [100,108-110]), and are also reported during MIA-induced knee osteoarthritis (in both the early and late phase of the disease) [111] (Table **4**). In some of these studies, bilateral injury was used to maximize the decrease in exploratory behavior [109,110]. Deficits in exploratory locomotion have also been observed during peripheral [112] and central neuropathy [113], postoperative (laparotomy) pain [24], visceral chemical irritation induced by acetic acid [114,115] and cyclophosphamide-induced cystitis [16]. This behavior can be quantified as either horizontal (ambulatory) or vertical (rearing) locomotion. Quantification of vertical activity is of particular interest when the lower limbs are injured, since this position maximizes the mechanical pressure of body weight on the injured hindlimbs (Table **4**) (e.g. [109,111,112]). A decrease in exploratory behavior has been pharmacologically validated as a pain outcome in all the models mentioned above with the exception of cyclophosphamide-induced cystitis (see Table **4**).

Another innate behavior that has been explored is burrowing. Laboratory rats, mice, hamsters and gerbils are fossorial animals, and therefore naturally burrow to hide from predators [116]. This innate behavior was recently used to detect the effects of pain on general condition in rodents. Burrowing behavior decreases clearly during inflammatory [117], neuropathic [117] or postsurgical (laparotomy) pain [118]. Burrowing deficits during these pain states were reversed by known analgesic drugs, validating this outcome as a pain measure suitable for detecting drug-induced analgesia [117,118] (Table **4**).

#### 3.2.2 Interference by Pain in Motivated Behavior: Wheel Running

More recently, motivated locomotor behavior has been targeted to detect voluntary changes in locomotor activity in rodents due to pain and its response to analgesics. Wheel running is an appetitive stimulus, and under repeated stimulation rodents reinforce this behavior, which makes it suitable for repeated measures after a training period. Locomotion in running wheels has been typically used in the past to measure voluntary physical activity in rodents [119], and it has been used more recently as an indicator of the interference by pain in appetitive physical activity. It has been shown that chemical irritation by acetic acid strongly decreased wheel running, and this effect was reversed by morphine [120]. In a recent study, we reported that bilateral hindpaw inflammation induced a marked decrease in wheel running, and the interference by inflammation on this activity was reversed by a variety of analgesic and anti-inflammatory drugs [52]. In addition, wheel running is also impaired during unilateral MIA-induced osteoarthritis, although to a limited extent [121] (Table **4**).

Since all these behaviors are much more complex than the standard reflexive withdrawal response, the interpretation of the experimental data is also less direct. Decreases in exploratory locomotion, burrowing or wheel running during a painful condition might simply be the result of the avoidance of acute pain episodes evoked by movement or by impact of the injured area on the floor during physical activity. However, such decreases would also be expected to be influenced by loss of motivation to perform the activity due to ongoing pain. In addition, taking into account that at least wheel running is widely considered to be a recreational activity for the rodents [119], decreases in this particular behavior might reflect some emotional aspects of pain, such as anhedonia. Therefore, the phenotype observed might be more complex than a simple measure of the physical capabilities of rodents during pain. In any case, since the performance of exploratory locomotion, burrowing or wheel running is not forced, it depends on the rodents whether and to what extent they perform them. Therefore, the recovery of normal behavior by analgesic treatment might be akin to the self-assessment by rodents of the efficacy of the intervention.

#### 3.2.3 Common Characteristics of Spontaneous Innate and Motivated Behaviors as Pain Indicators

Drugs might decrease the occurrence of reflexes without showing a true analgesic effect by affecting general motor activity; i.e. motor deficits can suppress paw withdrawal without eliciting analgesia [104]. In these nonreflexive outcome measures, a drug of these characteristics would never remove the interference by pain in the target behavior (i.e. it would not increase exploratory behavior, burrowing or wheel running) [52,114,117], and therefore its effects will not be confused with a false analgesic-like effect. On the other hand, because the return towards baseline motor activity is interpreted as an analgesic-like effect, drugs that enhance locomotion were thought to result in false positives. In a test of this hypothesis, the nonanalgesic stimulant caffeine increased horizontal exploratory locomotion in both injured and uninjured animals, although this effect can be readily detected in sham animals [114]. We used the same nonanalgesic control in wheel running activity in mice with inflammatory pain, and found that it did not result in an improvement in the outcome. Therefore, stimulating general activity did not lead to false positive results in this test [52]. Interestingly, amphetamine, although it increased horizontal exploratory locomotion, was unable to increase vertical activity in animals with hindlimb inflammation, and therefore did not result in a false analgesic-like effect in this measure [109].

Interestingly, some drugs showed biphasic effects on these outcomes. Although 30 mg/kg gabapentin was able to restore normal burrowing behavior during peripheral neuropathy, a higher dose (100 mg/kg) that induced prominent sedation (and decreased the target behavior in sham animals) did not have any ameliorative effect on burrowing in neuropathic animals. Similarly, doses of the opioid analgesic morphine that interfered with vertical movement or wheel running activity were unable to ameliorate the deficit in these measures induced by inflammation or osteoarthritis; i.e. doses of morphine that induced clear side effects did not show analgesic-like effects on nonreflexive outcomes [52,109-111]. Therefore, doses of analgesic drugs that also induce clear adverse effects would not result in an improvement in the outcome (Table **4**).

Finally, standard rodent pain measures are prone to experimenter bias (e.g. [8,122]), and are susceptible to confounding effects related to human–animal interactions [123,124]. The measurement of these outcomes can be fully automated, and there is no need for the presence of the experimenter near the evaluation area (except to administer the drug). Therefore, these potential confounders are mitigated in these outcomes.

## 4 EVALUATING MOTIVATIONAL ASPECTS OF PAIN AND PAIN RELIEF IN RODENTS

Pain relief can be obtained by different strategies depending on the source of the pain. When the source of pain is external, pain relief can be obtained simply by escaping from the noxious stimulus, which is an innate and universally present defensive mechanism [126]. Pain is a motivational (aversive) experience, and therefore once the source of pain is recognized, learning processes are triggered and will lead to avoidance of the noxious stimulus [11]. However, when pain comes from within the body, as during a painful disease (and is therefore inescapable), this strategy becomes invalid. During chronic pain conditions, appropriate pain medications provide relief to patients. Therefore, during a pain state (even if it is simply a mild headache), humans can actively seek an analgesic to provide pain relief. The pleasantness of the relief obtained with the analgesic is consistent with the definition of a reward, and therefore, analgesia has motivational (rewarding) properties when a pain state arises [127].

In studies undertaken to explore these motivational aspects of the pain phenotype, several approaches have been used in experimental animals to test the effects of drugs on pain aversion and the rewarding properties of analgesia as measures of the efficacy of analgesics.

### 4.1 Assessing Thermal Aversion in Rodents

Although most of the behaviors examined above probably have a strong component of pain avoidance, methods have been developed to specifically measure escape and avoidance of a recognized uncomfortable or painful stimulus. These experiments have used thermal stimulation, which provides fine control of the intensity of the sensory stimulation. Thermal pain avoidance can be readily measured using a hot or cold surface with an adjacent thermally neutral compartment which provide an escape option (e.g. [128]), or by using two adjacent hot or cold plates set at different temperatures, in which the occupancy of each plate and the number of crosses between them can be recorded (e.g. [129]).

These methods were initially used to determine nociceptive pain in uninjured rats [128,130-132]. Interestingly, at a mildly noxious temperature (44 °C), latencies to the first escape response were considerably lower than lick or guard latencies [133], indicating that animals are suffering discomfort long before the reflexive response occurs. One interesting finding from the pharmacological characterization of this outcome was that morphine attenuates operant escape at lower doses than the reflexive response [130-132] (Table **5**). This finding is consistent with the observation by Ercoli and Lewis in 1945 that in response to thermal stimulation (light beam) directed at the back of the animal, the reflexive (skin twitching) response was less affected by morphine than the latency to escape from the pain source [134]. Similarly, other early studies that tested the effects of morphine with the conventional hot plate found that morphine altered the jump response more strongly than the standard licking response [135]. These data show that different behavioral responses to the same thermal stimulation probably reflect different aspects of the pain experience, and point to the importance of the outcome for assessing the effects of potential analgesic compounds.

In addition to the evaluation of acute pain, the assessment of thermal avoidance was shown to be sensitive enough to reveal cold aversion in animals treated with the cold sensitizer icilin [136] and in neuropathic animals [129,137]. In the latter experiments, gabapentin used as a control antineuropathic drug was able to reverse the enhanced cold aversion induced in the CCI [129], a result that demonstrated the usefulness of this approach for drug testing (Table **5**). As described in the preceding section on experiments that involve innate and motivated behaviors, drugs that alter exploratory behavior (e.g. sedative drugs) can affect the results, since the time spent in the testing area may not correlate with the actual sensory perception of the rodent. In fact, amitriptyline at a dose that induced obvious motor effects was unable to significantly ameliorate the enhanced cold aversion in neuropathic animals [129] (Table **5**). Drug-induced motor effects that alter the target behavior can be easily monitored by measuring locomotor activity in the absence of thermal stimulation (i.e. the number of crossings between two neutral plates). Controls in the same environment would help to discriminate between the actual analgesic effects and the drug-induced sedative effects or motor impairment (see Table **5** for a summary of the results).

More recent studies have adapted thermal avoidance methods to the study of orofacial pain. Animals were trained to place their face against a stimulus thermode to access positive reinforcement (sweetened condensed milk). This presents a conflict between the positive reward and tolerance for thermal stimulation. Work with this experimental paradigm showed that heated thermodes induced a strong aversion in animals after the administration of carrageenan or capsaicin bilaterally in the mid-cheek region of the face [138,139]. Avoidance of contact with heat was fully reversed by morphine at a dose as low as 0.5 mg/kg [138,139]. The same procedure can be used to detect cold hypersensitivity in the orofacial region, by simply changing the temperature of the thermode. Increased cold sensitivity was detected after the administration of the cold sensitizers icilin and menthol [136,140] (Table **5**).

### 4.2 Conditioned Place Aversion and Preference Induced by Pain and Analgesia

Conditioned place preference is classically used in psychopharmacology to measure the rewarding or aversive motivational effects of objects or experiences (e.g., drugs of abuse). It is based on the animal’s choice between two different sides within the test chamber with well defined distinct environments after a conditioning phase in which the rodent associates the experimental manipulation (typically a drug) with one of the environments. In later sessions, if the manipulation has motivational significance, the animal’s choice will translate into a change in the time spent in the area associated with the experimental manipulation. A decrease in the time spent in the chosen environment is considered to indicate aversion to the experimental manipulation (conditioned place aversion). On the other hand, increases in the time spent in that area are considered to reflect the rewarding properties of treatment (conditioned place preference) [141]. This approach has been used in rodents to determine the efficacy of analgesic treatments by evaluating the association between pain (aversion) or analgesia (reward) and a specific environment.

It was shown that rodents exhibit conditioned place aversion to a chamber associated with formalin or acetic acid administration [142-145]. Conditioned place aversion was suppressed by timolol and the known analgesic clonidine at doses that did not decrease the nociceptive responses to formalin or acetic acid [144,145]. Further experiments in a more clinically relevant pain model found that rodents exhibited place aversion to a chamber associated with carrageenan-induced inflammation [146,147]. Conditioned place aversion during chronic inflammatory or neuropathic pain can also be induced by applying mechanical stimulation. In these experiments, two different locations within the test chamber were differentially associated with von Frey stimulations in the injured or noninjured paw. As a result, the animals developed conditioned place aversion to the side of the test chamber associated with mechanical stimulation in the injured paw [148-154]. It seems clear therefore that the pain experience has a motivational significance for the rodents, and they develop aversion to the pain-associated environment, reflecting the affective dimension of pain. Interestingly, drug sensitivity to the analgesic-like effect of drugs in these conditioned place aversion paradigms was much higher than for the reflexive outcomes measured by standard techniques in inflammatory or neuropathic conditions, and for a wide variety of drugs [146,147,149,152-154] (see Table **6** for a summary). A drug-induced decrease in pain aversion was observed upon the administration of timolol, clonidine or morphine in several brain structures such as the central nucleus of the amygdala, the bed nucleus of the stria terminalis (a major output pathway of the amygdala) and the anterior cingulate cortex, and at drug doses that did not affect reflexive pain-like responses [143-145,151] (Table **6**). Because these brain structures are thought to be involved in the affective dimension of pain [155,156], the findings indicate the involvement of this component of pain in the outcome evaluated.

Other authors, using the same experimental philosophy but the opposite approach, attempted to determine the rewarding properties of analgesic drugs in rodents during a pain condition instead of looking for the effects of treatments in pain aversion. It was shown that rodents with inflammatory, osteoarthritis, central or peripheral neuropathic pain can show conditioned place preference to an environment associated with successful analgesic treatment [61,85,157-162] (Table **6**), indicating that the rodents craved analgesic treatment. Since after receiving the treatment the animals can freely choose or reject the analgesic-associated environment, this is an indirect evaluation of the self-reporting effects of analgesic treatments on overall (including ongoing or spontaneous) pain. One of the most interesting findings of these experiments was that adenosine, a drug able to reverse mechanical allodynia in rodents, was unable to induce any conditioned place preference in rats with neuropathy rats [159]. Importantly, this is in agreement with the reported absence of effect of adenosine on overall pain in human patients [163], although it exerted antiallodynic effects [163,164]. Another interesting finding that dissociates reflexive hyperalgesia to overall pain was that a TRPV1 antagonist, at doses effective in thermal hypersensitivity, was unable to induce place preference in rats with inflammatory pain, showing that the treatment apparently did not ameliorate pain [160]. This does not imply that the reversion of sensory hypersensitivity does not account for the overall self-perception of pain, since the drugs used in these studies also reversed hypersensitivity [61,85,159,161], but instead suggests that this is probably not the only factor to consider in preclinical testing of analgesic candidates and the translation into useful, clinically relevant compounds.

Although these techniques are methodologically more complex and time-consuming than standard methods, they can provide valuable information about the effectiveness of drug treatments as an indirect measure of the self-reported analgesic efficacy of treatment. 

### 4.3 Self-administration of Analgesics

Self-administration techniques are typically used to determine the abuse potential of psychoactive drugs. This is the primary nonclinical approach to assess the reinforcing properties of novel compounds, and its predictive validity has been shown to be strong [166]. Efforts have been made to adapt this technique to the measurement of the reinforcing properties of analgesia during a pain condition.

Early experiments were conducted to determine if rodents are able to self-administer an analgesic when they are in chronic pain. In the simplest setting, two different drinking solutions can be offered to animals, one of them with an analgesic drug and a different one with a sweet palatable solution. Rodents usually prefer the sweet solution rather than the analgesic-containing solution. However, during CFA-induced inflammation, rodents increased their consumption of opioid analgesic fentanyl or the NSAID suprofen [167-169]. Similarly, rodents also increased their consumption of ibuprofen after surgical manipulation [170]. In contrast, during peripheral neuropathy, for which opioid analgesia is not as effective as for inflammation [171], rodents did not increase their consumption of fentanyl [168] (Table **7**). These results strikingly show that animals are able to decide if an analgesic treatment is having the desired effect or not. Although these experiments are highly informative, they are not very common in preclinical research, since their design is complex and several factors need to be taken into account when the results are interpreted, such as the rodent’s basal preference for solutions, the reinforcing properties of the drug in the absence of pain, and the balance between analgesia provide by the analgesic solution and the positive reward obtained from the palatable solution, which might change during a pain state as will be described in section 5.2. below.

Recent experiments used a much more complex setting in which animals could obtain a bolus of analgesics by either pressing a lever with their paws or by nose-poking. With this methodology it was shown that neuropathic rats self-administered more clonidine spinally than control animals [172], clearly indicating that the animal found the treatment to be rewarding. In a similar setting, it was recently shown that rodents increased their self-administration of two promising candidate analgesics: a CB2 agonist ((R,S)-AM1241) [173] and a sigma-1 antagonist (S1RA) [174] at a dose able to attenuate sensory hypersensitivity during peripheral neuropathy. These results clearly show that CB2 agonism and sigma-1 antagonism are positively reinforcing stimuli during neuropathic pain, but not under normal conditions, adding further evidence of the potential use of CB2 and sigma-1 receptors as therapeutic pain targets [173,174] (Table **7**). Further experiments have used opioid analgesics with somewhat less clear results. During peripheral neuropathy, when the system was set to administer a low dose of opioids (purportedly insufficient to induce analgesia), animals decreased the rate of response [175]. Only doses of analgesics able to reverse mechanical allodynia maintained operant responding [175] (Table **7**). The interpretation of these results is complex, since the decrease in the rate of administration of a low dose of opioids might reflect anhedonia (the lack of interest or pleasure in responding to normally hedonic stimuli or experiences) induced by the pain condition, and the animals did not increase their rate of response compared to sham-operated animals even when the dose administered was enough to reverse mechanical allodynia [175]. Together, these results may reflect the poor analgesic efficacy of opioids during neuropathic pain, along with emotional changes during chronic pain.

Assessing the self-administration of analgesics is probably, in methodological terms, the most complex and time-consuming of the techniques described in this review, and therefore not appropriate for screening to identify potential analgesics. However, these tests can provide extremely useful information about the effectiveness of a selected drug treatment, since animals self-administer the drug (unlike all other behavioral tests).

## 5 PAIN-INDUCED EMOTIONAL DYSFUNCTION: ANXIETY, DEPRESSION AND SLEEP DISTURBANCES

There is a substantial body of human research supporting the association of chronic pain with high levels of emotional distress, particularly depression and anxiety [176]. This is not surprising since prolonged pain can be conceptualized as a complex form of stress [177,178], and stress plays a pivotal role in the development of both depression and anxiety [179-183]. Persistent pain, together with the physical impairment often associated with pain conditions, are likely to produce high levels of stress which greatly contribute to anxiety and depression processes (e.g. [184,185]). In addition, depression and anxiety in human patients are often comorbid with sleep disturbances [186], and this has been repeatedly observed in human pain patients [185,187-190]. Sleep disturbances have been associated with pain severity (e.g. [189,190]), nocturnal pain episodes (e.g. [190]), physical disability (e.g. [185]) and its consequent psychological disturbances (e.g. [185,187,190]). Together, these problems have a substantial negative impact on the daily activities of patients.

### 5.1 Development of Anxiety-like Behaviors During a Pain Condition

The experimental paradigms used to study anxiety-related behaviors consist of exposing animals to aversive stimuli capable of causing an “anxious state” while observing and quantifying their behavior. Tests to assess anxiety in rodents differ in the stimuli they use to induce this state. These stimuli include open spaces, illuminated areas and environmental or social novelty. The open-field, elevated plus/zero maze and the light-dark tests are based on a natural conflict between the drive to explore a new environment and the tendency to avoid a potentially dangerous area (open space or brightly-lit area), and the time spent in the “dangerous” and “safe” areas can be measured [191,192]. Social interaction tests are different since the stressor is a live conspecific, and the outcome measure is the approach of the animal being tested to a novel (stressor) or familiar animal [191,192]. Marble-burying behavior has also been extensively used to measure anxiety in rodents. A high level of marble-burying is thought to indicate anxiety caused by the novelty of marbles [193].

These behavioral evaluations have long been used in preclinical studies to test for possible anxiolytic or anxiogenic compounds and to record effects of experimental manipulation on anxiety behaviors [194-201]. It is only recently, however, that these tests have been used in animals under a pain condition. These tests have been used to detect possible anxiety behaviors in rodents during inflammation or peripheral neuropathic pain, as well as after hindpaw incision or chemical irritation by intraperitoneal acetic acid. The findings reported to date are contradictory, and therefore it is not clear whether rodents develop anxiety-related behaviors as a consequence of pain in these models. In fact, one study reported that animals under peripheral neuropathic pain showed a decrease in anxiety behavior [202]. A summary of these studies is shown in Table **8**.

Pharmacological experiments were carried out in some of these studies. Anxiety-related behaviors during neuropathic pain were reversed by morphine, gabapentin and duloxetine at doses that did not alter the behavioral response in pain free animals. This suggests that the suppression of pain induced an improvement in the emotional state of the animals, which in turn led to a decrease in anxiety behavior [112,203,204]. In addition, the enhanced anxiety-like behaviors during peripheral neuropathy were almost fully suppressed by imipramine, milnacipran, paroxetine and midazolam, although their effects in neuropathic sensory hypersensitivity were modest or absent [204,205]. To date, only one study has explored the effect of the antineuropathic drug gabapentin in a model of nontraumatic peripheral neuropathy induced by *Varicella zoster* virus, and found that this drug was unable to ameliorate deficits in the open field test in animals with neuropathy [203] (see Table **8**). Morphine was also shown to decrease anxiety-like behaviors (elevated plus maze) during chronic inflammation and after hindpaw incision [206,207], and gabapentin was also reported to be active in hindpaw incision-induced anxiety [207]. One experiment involving inflammatory pain-induced anxiety showed that the corticotrophin-releasing hormone receptor 1 (CRF1) antagonist NBI27914 successfully reversed inflammation-induced anxiety behavior [208]. 

Interestingly, some of these treatments (paroxetine and the CRF1 antagonist NBI27914) have been shown to be effective in anxiety-like behaviors when administered in structures of the limbic system (the amygdala and cingulate cortex) [205,208] (see Table **8** for details). These structures are thought to play a role in both anxiety [209] and the affective component of pain [155,156], and therefore both pain relief and anxiolytic activity might contribute to their effects. On the other hand, benzodiazepines (etizolam or diazepam), which do not have analgesic effects in inflammatory pain [210], decreased inflammation-induced anxiety (elevated plus maze and light-dark test) without any apparent decrease in sensory hypersensitivity. These results suggest that anxiety-related outcomes might be affected indirectly by analgesic drugs (through analgesia) or directly by anxiolytic drugs [206,211] (Table **8**).

### 5.2 Development of Depressive-like Behaviors During a Pain Condition

The most popular experimental paradigms to study depressive-like behaviors are the forced swimming test [224] and tail suspension test [225]. These tests are based on behavioral adaptations to inescapable, highly aversive situations (despair-based measures). The animals are placed in an uncomfortable, stressful position (water tank or suspended by the tail), and after an initial period of attempting to escape, they typically assume a passive posture (despair-reaction). Both tests are widely used to assess the antidepressant-like activity of compounds and depressive-like behaviors [224-226]. A different approach is to evaluate anhedonia, a cardinal symptom of depression [227], as an indicator of the animal’s emotional state (reward-based measure). Rodents show a natural preference for the sweet taste [228], and this preference can be quantified by giving them a choice of two bottles containing water or a sweet (sucrose or saccharine) solution. A reduction in the consumption of the sweet solution compared to water is considered to represent anhedonia in rodents, and it has been widely shown to be sensitive to different pharmacological and environmental manipulations in standard animal models of depression [229-231]. A different type of depressive-like outcome is the decrease in grooming behavior after a sucrose solution is squirted onto the animal’s fur (splash test) as an indicator of the effects of depression on self-care [232].

As in models of anxiety, the results are diverse and contrasting, although with a somewhat higher preponderance of positive alterations (see Table **9** for a summary of the studies). A few pharmacological studies have shown that ketamine, desipramine and oxytocin, which are known to ameliorate both depression and pain [233-236], were effective in reversing depressive-like behaviors (forced swimming test) in rats with neuropathy, and that the effects were seen at lower doses that those needed for alleviation in sensory hypersensitivity [218,237,238]. The synthetic CB2-selective agonist GW405833 was shown to be effective reversing both depressive-like behaviors and sensory hypersensitivity [238].

Sucrose and saccharine preference tests have recently been used to investigate anhedonic-like behavior in different pain models, with contrasting results (see Table **9** for a summary of the studies). Only three studies up to now showed pain-induced anhedonic-like behaviors. We recently reported that a 6-hour decrease in the preference for saccharine was observed in mice after the intraperitoneal injection of acetic acid, and that this preference was fully restored by the analgesic ibuprofen but not by the general stimulant caffeine [115]. Two of these studies showed anhedonic-like behavior during a chronic pain condition (peripheral neuropathy) [174,237]. Interestingly, ketamine, which is known to exert both antidepressant and analgesic properties [234,235], completely reversed the anhedonic-like behavior at doses that did not alter sensory hypersensitivity [237]. The candidate antineuropathic drug S1RA (a sigma-1 antagonist) was also very recently shown to be able to reverse neuropathic pain-induced anhedonic-like behavior [174] (Table **9**).

### 5.3 Possible Confounders and Sources of Variation of the Evaluation of Psychological Distress

In spite of the positive data, it is difficult to explain the inter-study variations: some studies reported a marked effect on anxiety-like or depressive-like behaviors, whereas others found no behavioral effect at all (see Tables **8** and **9**). Since the emotional state of rodents plays a major role in these outcomes, housing (environmental enrichment, noise, etc.) and handling conditions are expected to influence these behaviors. This assumption is supported by a recent study showing that psychological changes were potentiated under stressful conditions [217] or alleviated when mice with neuropathy were housed under pair conditions (social support) [218]. The test modality seems particularly important in tests of anxiety, since in some reports animals showed anxiety-like behaviors in some paradigms but not in others [112,206]. Different tests may therefore look at only partially overlapping constructs [191], although it is not clear how applicable this hypothesis is to human pain patients. Another source of variability might be repeated pain testing in the same animals, since pain is a known stressor [177], and in some studies pain might be measured by additional sensory stimuli (hypersensitivity) in the same animals. In addition, it was recently reported that the emotional deficits differed between animals with peripheral neuropathy in the left or right hindpaw. Animals with traumatic nerve injury in the left hindlimb, but not in the right hindlimb, had a more clearly anxiety-like profile [162]. Therefore, the side of the injury is another possible variable to consider. Other perhaps more obvious considerations to take into account are that the pain (or pain-induced disability) induced in current pain models might simply be insufficient to induce consistent psychological changes in rodents, or simply that rodents do not suffer marked psychological changes in response to a pain condition as humans do.

### 5.4 Pain-induced Sleep Alterations

Sleep alterations during a pain condition can be assessed by measuring several parameters, including time awake, latency to sleep, sleep time, number of arousals during sleeping time, and latency and time spent during REM or non-REM sleep. Sleep alterations have been consistently reported during inflammation in the hindlimb [239-242] or the orofacial region [243-245]. Only one study reported sleep alterations during MIA-induced osteoarthritis, which were produced mainly in the latter neuropathy-like phase [246]. The occurrence of sleep alterations during peripheral neuropathy has not been reported consistently, and contradictory results can be found in the literature. Although one study showed no effects of peripheral neuropathy in sleep [247], a second study showed obvious sleep alterations although mostly limited to the first week after injury [248]. These discrepant results might be explained by the reported high variability between individuals, since only a limited subpopulation of rats showed alterations in sleep (45% showed no effect and 25% showed only transient effects) [249]. These studies are summarized in Table **10**.

A few pharmacological studies have been performed during adjuvant induced inflammation. Acetaminophen, indomethacin, the selective COX-2 inhibitor etoricoxib and the nitric oxide synthase inhibitor L-NAME (not used in clinical studies) were effective in ameliorating sleep disturbances [241,243-245], but acetylsalicylic acid showed a detrimental effect [241]. Interestingly, all these drugs with the exception of indomethacin affected sleep in noninjured animals [241,243-245], (see Table **10** for a summary of these studies). More pharmacological studies are needed to determine the possible usefulness of this outcome for testing candidate analgesics. Only one study showed the effects of drugs on sleep during neuropathic pain. A GABA transporter (GAT-3) inhibitor was shown to ameliorate sleep disruption during neuropathy when injected into the intracingulate cortex. Interestingly, the benzodiazepines midazolam and zolpidem, but not pentobarbital, showed much smaller effects in animals with neuropathy than in pain-free animals, clearly pointing to an effect on GABAergic neurotransmission during neuropathy [250].

## 6 WHAT HAVE WE LEARNED FROM NONREFLEXIVE OUTCOMES?

Individuals with pain differ from individuals without pain not only in how they “feel” but also in how they “behave”, and this is applicable to both humans and rodents. Although preclinical pain research, and hence the development of new analgesics, is hampered by the inability of rodents to communicate verbally, here we show that with the proper tools and knowledge, basic pain researchers can detect a wide range of aspects of the pain phenotype in rodents which closely resemble the human pain phenotype. The successful use of these nonstandard outcomes in several pain models are summarized in Table **11**.

### 6.1 Do the Current Models Induce Disability in Rodents that Parallels Pain Conditions in Humans?

It is worth noting that a number of the experiments summarized here used bilateral injuries to obtain a reliable influence of pain on the target behavior. These outcomes include grip strength (Table **2**), home cage behavior (Table **3**), exploratory locomotion (Table **4**), wheel running (Table **4**) and thermal avoidance (Table **5**). Since rodents are quadrupeds, a single hindlimb injury may not induce the same level of pain-induced disability as an injury in a human arm or leg. Therefore, a single limb injury may be insufficient to consistently drive complex changes in the behavior of the rodents. The use of bilateral injuries might improve the determination of anxiety- and depressive-related behaviors in rodents under chronic pain, as well as sleep alterations during neuropathy in rodents, which so far have yielded inconsistent results (Tables **8**-**10**), and would make it possible to reliably test new therapies against this part of the pain phenotype with relevance to humans. Although a single-limb injury is obviously sufficient to study sensory hypersensitivity, it might not mimic the complexities of the human pain phenotype closely enough in a number of outcomes of interest. We understand that for some researchers this issue can raise ethical concerns. If hypersensitivity is the only measure needed for research purposes, performing bilateral injuries would be unnecessary. However, if the aim is to study complex behavioral changes produced by pain, bilateral injuries might be needed.

### 6.2 Sensitivity of Nonreflexive Outcomes to Analgesic Drugs

Several of the nonreflexive outcomes of interest share an extraordinary sensitivity to drug-induced analgesic-like effects in comparison to standard reflexive outcomes. As shown in the summary tables presented here, this enhanced sensitivity to drug-induced analgesia is seen for known analgesics of different classes, such as opioids and NSAIDs, and in a variety of outcomes. Morphine and other opioids are known to ameliorate the affective component of pain [155,252]. Since many of these tests have a motivational and affective component, this effect might provide an explanation for the enhanced sensitivity to morphine of the decrease in exploratory and running wheel activity induced by inflammation ([109, 52], Table **4**), thermal avoidance ([130-132], Table **5**) and place aversion conditioned by pain ([146,152,154], Table **6**). However, the exclusive effects on the affective component of pain do not explain the greater sensitivity to intrathecal morphine of the pain-induced decrease in exploratory locomotion [125], or the enhanced analgesia seen with NSAIDs such as aspirin or ibuprofen in the recovery from weight bearing asymmetry ([56,57], Table **1**), exploratory locomotion ([109,125], Table **4**), burrowing ([117], Table **4**), wheel running [52] (Table **4**) and pain-induced place aversion ([147,153,165], Table **6**). Therefore, the high sensitivity to analgesics does not appear to be a peculiarity of these nonreflexive outcomes or specific drugs, but reflexive measures seem to be particularly resistant to drug-induced analgesia. Hence drug treatments can successfully induce analgesia without having analgesic-like effects on reflexive outcomes such as von Frey test results.

Several other drugs, including benzodiazepines (midazolam), tricyclic antidepressants (imipramine and desipramine), SNRIs (milnacipram), SSRIs (paroxetine), the NMDA receptor antagonist ketamine and the hormone oxytocin improved anxiety- or depressive-like outcomes without affecting sensory hypersensitivity, particularly during neuropathy [204,205,217,218,237,238] (Tables **8** and **9**). Although the actions of these drugs might be explained by their known antidepressant or anxiolytic properties, all these drugs are also known to have antineuropathic properties [233-236,253-257]. Pain reduction can also lead to an improvement in the emotional state of animals under a pain condition [204], and taking into account the broadly greater sensitivity of nonreflexive outcomes for the detection of drug-induced analgesia, the ameliorative effects of these drugs might be due, at least in part, to their analgesic actions (even if they do not ameliorate mechanical allodynia in von Frey tests) and not only to their anxiolytic/antidepressant properties. Further experiments with these compounds and different nonreflexive outcomes are needed to determine whether these drugs induce their ameliorative effects exclusively through their anxiolytic/antidepressant activities or whether true analgesia is also involved.

Among studies that compared drug sensitivity measured with reflexive and nonreflexive outcomes, only four reported similar sensitivities with both approaches for certain drugs [109,110,165,238], and only one study showed a much lower sensitivity to drug-induced analgesia in a nonreflexive outcome compared to a reflexive response. Specifically, morphine was shown to be 56-fold less potent in reversing the depression in locomotion induced by the intraperitoneal injection of acetic acid compared to acetic acid-induced writhing [114] (Table **4**). This finding might be explained by the fact that at the time the effects of morphine were evaluated, mice were actively writhing, so morphine was required to reverse the decrease in both the reflexive and nonreflexive responses occurring at the same time. Therefore, although with a few exceptions, the high sensitivity of these outcomes to drug effects appears to be an intrinsic quality of these measures. Tests that explore nonreflexive outcomes were designed for diverse purposes, including the study of ongoing or spontaneous pain, pain-induced physical disability, overall well-being in rodents, the affective component of pain or even psychological disturbances induced by pain. Although these outcomes look at different facets of the pain phenotype, they all evaluate the consequences of pain for a nonreflexive behavior. 

Work based on pharmacokinetic and pharmacodynamic analyses of the analgesic effects of drugs in humans and rodents found that the effective doses in many animal models of pain overpredict the clinical doses necessary to relieve pain in humans [12,258]. Therefore, the enhanced drug sensitivity of nonreflexive outcomes might be better at predicting efficacy in “human” clinical pain than the standard outcome measures of hypersensitivity. A similar rationale has been invoked in the past for the differential sensitivity to analgesics of acute and tonic (formalin-induced) pain – a view that marked the transition from the study of pain as an acute phenomenon to more clinically relevant models (reviewed in [13]). The use of nonreflexive outcomes might constitute the next step in efforts to appropriately define analgesic efficacy in rodents, although further research is obviously needed to understand the meaning of differential drug sensitivity between nonreflexive and reflexive outcomes. Such studies might be facilitated by referring to dose-response curves for known analgesics in several nonreflexive and reflexive outcomes, although these curves are often not obtained. The profile of the reflexive and nonreflexive behavioral changes induced by standard effective treatments in rodent pain models would aid in the study of potentially useful new analgesics by facilitating comparisons to the behavioral effects of reference compounds.

### 6.3 Pain Specificity of Nonreflexive Measures

The goal of analgesic drug development is to test not only therapeutic efficacy, but also toxicity, so that therapeutic indices can be accurately estimated. Since the resulting overall behavior is a balance between the therapeutic (analgesic) and adverse (nonanalgesic) effects induced by the drug tested, several of these nonreflexive measures are often able to dissociate drug doses that have analgesic efficacy from those that induce side effects affecting the target behavior. For instance, morphine, an analgesic par excellence used for centuries in pain management, has biphasic effects on many of these outcomes, including grip strength ([92], Table **2**), exploratory activity ([109-111], Table **4**), wheel running ([52], Table **4**) and pain-induced place aversion ([165], Table **6**). Its bell-shaped dose-response curve is thought to reflect the side effects of this drug, which can alter the behavior of rodents at moderate or high doses. Other examples of known analgesic drugs which do not improve nonreflexive outcomes at moderate or high doses are gabapentin in burrowing behavior in neuropathic animals ([117], Table **4**), amitriptyline in cold aversion in animals with neuropathy ([129], Table **5**), or tramadol in exploratory behavior in osteoarthritis pain ([111], Table **4**). Therefore, the same outcomes can be used to detect drug-induced analgesia in injured animals and acute toxicity in noninjured animals; and doses of analgesic drugs that also induce clear adverse effects will not improve the behavioral outcome. Predicting clinical efficacy involves the appropriate identification of side effects at analgesic doses, and in this sense the detection of drug-induced side effects by these outcomes is enormously advantageous and informative. However, the detection of non-analgesic drug effects in these behavioral tasks also points to the lack of pain specificity of these outcomes (i.e., although these outcomes can be altered as a consequence of pain, they are not direct measures of pain as is paw withdrawal in response to noxious sensory stimulation).

If we take into account that most of these tests (with the notable exception of weight bearing asymmetry) were designed for many other purposes (e.g. the study of neurological disturbances, muscular strength, drug addiction, anxiety, etc.), it seems clear that they can be affected by several factors other than pain. For instance, drugs that increase locomotion are expected to recover the pain-induced decrease in locomotion without exerting real analgesic effects [114]. Similarly, drugs with anxiolytic properties can be expected to improve anxiety-like behaviors during a painful condition, but not necessarily pain itself (e.g. [206,211]), and drugs with sedative properties might improve sleep during a painful condition even if they are devoid of any analgesic property. Understanding and recognizing the advantages and limitations of these and most standard outcomes should lead to their use in a rational and integrated manner, with the aim of obtaining the most complete information possible about the biology and possible clinical usefulness of candidate analgesics.

### 6.4 Should we Switch from Reflexive to Nonreflexive Outcomes in Preclinical Pain Research?

Reflexive outcomes are not without obvious notable qualities. They can be used to evaluate sensory hypersensitivity, which is undoubtedly a feature of chronic pain [29,259]. One of the most important qualities of reflexive outcomes is their pain specificity. This is not to say that nonanalgesic drugs are unable to alter these behaviors in spite of their pain specificity, since it is known that sedative drugs or muscle relaxants can suppress a pain-induced reflex even if they are devoid of analgesic efficacy [13,104]. Experiments based on reflexive outcomes can be performed relatively quickly and in some cases with the same animals (e.g. [27,32]). This speed of data acquisition is useful not only to save time and money for researchers, but also because the same animals can be used in time-course studies of a drug’s effects, and this is useful to obtain information about the kinetics of a candidate analgesic. Reflexive outcomes are also ideal for mechanistic studies, since mechanical or thermal stimulation can be applied to the animals and the behavioral phenotype can be related to specific cellular or molecular responses. The most important quality that argues in favor of reflexive outcomes for preclinical pain research is that all known analgesic drugs clinically used in humans are able to decrease reflexive outcomes in rodents, so it is probably a necessary quality for a clinically useful drug to decrease most standard rodent pain outcomes. However, if reflexive tests are used as the only behavioral endpoint, they might be insufficient to reflect the pain experience holistically. For instance, adenosine ameliorates neuropathic mechanical allodynia in both human patients [163,164] and rodents [159], but this is not enough for patients to consider the drug successful in relieving overall pain [163], and does not induce craving for the treatment in rodents (i.e. it is not considered a successful treatment) [159]. In fact, although current analgesics are often highly effective in reversing reflexive hypersensitivity in rodents, their efficacy in humans is known to be limited [e.g. 260]. Therefore, targeting mechanical allodynia exclusively is insufficient to obtain irrefutable evidence that a given drug successfully ameliorates the overall status in either rodents or humans.

On the other hand, several of the nonreflexive outcomes described here can be challenging for researchers. For example, tests of pain-induced conditioned place aversion, sleep architecture analysis and operant analgesic self-administration are excessively time-consuming or methodologically complicated. Some methods such as exploratory activity measures are unsuitable for the repeated evaluations necessary to determine the time course of drug effects, because of the loss of novelty of the environment (needed to boost exploratory activity). Other methods such as sleep analysis, home cage locomotion, and to a lesser extent wheel running require long recording periods to obtain reliable measures and are therefore unsuitable for determining the analgesic efficacy of a drug at concrete time points. In addition, operant analgesic self-administration experiments are methodologically complicated in rats and even more difficult to perform in mice. Moreover, unlike von Frey or Hargreaves tests to measure sensory hypersensitivity, it is difficult to relate nonreflexive outcomes with a specific feature of the pain phenotype (allodynia, movement-evoked pain, ongoing or spontaneous pain or the affective and motivational components of pain) because these outcomes might act in conjunction, a characteristic that makes mechanistic studies difficult. Although nonreflexive outcomes might provide a more realistic reflection of pain and its consequences, most of them lack pain specificity as noted in the preceding section. Therefore, the information they provide is qualitatively different from that obtained with reflexive outcomes, and should be considered complementary in nature. Since the information obtained and possible confounders are different, we believe that a drug which improves both reflexive and nonreflexive behaviors might be more likely to be clinically useful for the treatment of pain than a drug that acts solely on mechanical allodynia or exclusively on a nonreflexive behavior. Therefore, to better understand the pharmacological profile of candidate analgesics, it would be wise to combine these methodologies with the most conventional tests for nociception instead of using them as the only endpoint.

## 7 CONCLUSIONS

In summary, we have reviewed the experimental evidence showing that rodents subjected to sustained pain change their behavior markedly. These behavioral consequences of pain can be detected by a battery of behavioral tests that are not limited exclusively to reflexive hypersensitivity. These behavioral responses attempt to translate the human pain phenotype from bedside to bench, and reflect the contribution of pain to physical and psychological functioning, and therefore to the quality of life of rodents. Although some of these behavioral tests could benefit from additional refinement, they can complement standard reflexive outcomes and provide rich information about novel pain targets or candidate analgesics. Some of these behavioral outcomes can be readily used to detect analgesic-like effects of candidate analgesics. It is to be hoped that translating outcomes from bedside to bench will improve the translation of new analgesics from bench to bedside.

## Figures and Tables

**Table 1.** Summary of Drug Effects on Postural Changes (Weight Bearing and Gait) During a Painful Condition

	Disease	Injury	Drug	Effect	Specie	Notes	References
**Weight bearing**	Inflammation	Carrageenan	Morphine	Effective	rat		[50,55]
			Diclofenac	Effective	rat		[50]
			Indomethacin	Effective	rat		[50]
		CFA	Rofecoxib	Effective	rat	More sensitive than tactile allodynia or hyperalgesia	[57]
			Naproxen	Effective	rat		[57]
			Diclofenac	Effective	rat		[57]
			Ketorolac	Effective	rat		[57]
			Ibuprofen	Effective	mouse		*
			Diazepam	Effective	rat	Only effective at doses that altered locomotion	[56]
			NS11394 (GABA_A_receptor-positive allosteric modulator)	Effective	rat	More sensitive than tactile allodynia	[56]
		LPS	Indomethacin	Effective	mouse		[58]
	Ostoarthritis (early phase)	MIA	Amitriptyline	Effective	rat		[59]
			Naproxen (chronic and acute)	effective	rat		[53,59,83]
			Diclofenac	Effective	rat		[60,73]
			Lidocaine (intraarticular)	Effective	rat		[60]
			Morphine (chronic and acute)	Effective	rat		[53,60,73]
			Dexamethasone (chronic and acute)	Effective	rat		[53]
			Duloxetine (chronic)	Effective	rat		[53]
			Duloxetine (acute)	Inactive	rat		[53]
			Acetaminophen	Effective	rat		[73]
			ABT-102 (TRPV1 antagonist)	Effective	rat		[77]
			Pregabalin	Inactive	rat		[53]
			Gabapentin	Unclear	rat		[59,73]
			Celecoxib	Unclear	rat		[53,59]
	Ostoarthritis (late phase)	MIA	Gabapentin	Effective	rat		[59]
			Amitriptyline	Effective	rat		[60]
			Morphine (chronic and acute)	Effective	rat		[53,60,84]
			Tramadol	Effective	rat		[84]
			Lidocaine (intraarticular)	Effective	rat		[60,61]
			Dexamethasone (chronic and acute)	Effective	rat		[53]
			Duloxetine (chronic)	Effective	rat		[53]
			Clonidine (i.t.)	Effective	rat		[85]
			AMG9810 (TRPV1 antagonist)	Effective	rat	Effective in moderate MIA-induced osteoarthritis	[61]
			Acetaminophen	Inactive	rat		[73]
			Duloxetine (acute)	Inactive	rat		[53]
			Celecoxib (acute)	Inactive	rat		[53,59,74]
			Celecoxib (chronic)	Unclear	rat		[53,59,74]
			Naproxen (chronic and acute)	Inactive	rat		[59]
			Indomethacin	Inactive	rat		[74]
			Pregabalin (chronic and acute)	Inactive	rat		[53]
			HC030031 (TRPA1 antagonist)	Inactive	rat		[61]
			Diclofenac	Unclear	rat		[61]
	Postoperative Pain	Paw incision	-		rat	Only slight weight bearing asymmetry was observed	[62]
	Peripheral nerve injury	CCI	Pregabalin	Inactive	rat		[64]
			Morphine	Inactive	rat	Effective at high doses	[64]
		SNL	Milnacipram (i.t.)	Inactive	rat	Borderline and fast effect	[82]
		SNI	Morphine	Inactive	mouse		[63]
			Gabapentin	Inactive	mouse		[63]
			EMLA cream (2.5% lidocaine, 2.5% prilocaine)	Inactive	mouse		[63]
**Gait**	Inflammation	Carrageenan	Morphine	Effective	rat		[70,71]
			Rofecoxib	Effective	rat		[70]
			Indomethacin	Effective	rat		[71]
	Peripheral neuropathy	Axotomy	-	-	rat		[71]
		PSNL	Gabapentin	Inactive	rat		[71]
			Duloxetine	Inactive	rat		[71]
		CCI	Gabapentin	Inactive	rat		[71]
			Duloxetine	Inactive	rat		[71]

Local administration or repeated treatments are indicated between brackets in the column headed “Drugs”, as well as the target of noncommercially available drugs identified by a code number.

* Unpublished data. i.t.: Intrathecal administration.

**Table 2.** Summary of Studies Focused on the Impact of Pain in Grip Strength Deficits

Disease	Injury	Drug	Effect	Specie	Notes	References
Inflammation (muscle)	Carrageenan (bilateral)	Levorphanol	Effective	rat		[89]
		Morphine	Effective	mouse		[91]
		Indomethacin	Effective	rat		[89]
		Dexamethasone	Effective	rat		[89]
		Dextrorphan	Effective	rat		[89]
		MK801 (NMDA antagonist)	Effective	rat		[89]
		WIN55,212-2 (CB agonist)	Effective	mouse		[90]
Cancer pain	Sarcoma implantation (bilateral)	Morphine	Effective	mouse		[91]
		WIN55,212-2 (CB agonist)	Effective	mouse		[90]
Osteoarthritis (late phase)	MIA	Morphine (low dose)	Effective	rat		[92]
		Morphine (high dose)	Inactive	rat	Sensitive to side effects induced by morphine	[92]
		Tramadol	Effective	rat		[92]
		Celecoxib	Effective	rat		[92]
		Diclofenac	Effective	rat		[92]
		Ibuprofen	Inactive	rat		[92]
		Indomethacin	Unclear	rat	Borderline significant effects	[92]
		Acetaminophen	Inactive	rat		[92]
		Duloxetin	Effective	rat		[92]
		Gabapentin	Effective	rat		[92]
		PD98059 (ERK kinase inhibitor, i.t.)	Effective	rat		[93]
		Lamotrigine	Inactive	rat		[92]
		Haloperidol	Inactive	rat		[92]
		ABT-102 (TRPV1 antagonist)	Effective	rat		[77]
		A-993610 (TRPV1 antagonist)	Effective	rat	Outcome improved with repeated dosing	[77]

The injuries were performed unilaterally unless otherwise indicated between brackets in the column headed “Injury”. Additional information about the drug treatment is included between brackets in the column headed “Drugs”. This information includes the target of noncommercially available drugs identified by a code number, local routes of administration, and use of a low or high dose (if differential effects depending on the dose were seen). i.t.: Intrathecal administration.

**Table 3.** Summary of the Effects of Pain on Home Cage Locomotion

Disease	Injury	Behavior Altered by Injury	Drug	Effect	Specie	References
Inflammation	CFA	No			mouse	[101]
	CFA	No			rat	[100]
	CFA (bilateral)	Yes			rat	[100,102]
	CFA	Yes	Acetaminophen*	Effective	rat	[99]
			Celecoxib*	Effective	rat	[99]
			Aspirin*	Inactive	rat	[99]
Osteoarthritis	MIA (bilateral)	Yes			rat	[103]
Peripheral neuropathy	CCI	Slightly			mouse	[101]
	SNI	No			mouse	[101]

The injuries were induced unilaterally unless otherwise indicated between brackets in the column headed “Injury”.

* Indicates that chronic drug treatments were performed.

**Table 4.** Summary of Studies Focused on the Effects of Pain in Innate (Exploratory Locomotion and Burrowing) and Motivated (Activity in Running Wheels) Behaviors

Outcome	Disease	Injury	Type of Locomotion	Drug	Effect	Species	Notes	References
**Exploratory behavior**	Inflammation	Carrageenan	Vertical	Ibuprofen	Effective	rat	Similar sensitivity to thermal hyperalgesia	[110]
				Diclofenac	Effective	rat		[110]
				Morphine (low dose)	Effective	rat		[110]
				Morphine (high dose)	Inactive	rat	Sensitive to side effects induced by morphine	[110]
				Gabapentin	Inactive	rat		[110]
				Duloxetin	Inactive	rat		[110]
		CFA	Horizontal	Morphine	Effective	rat		[100]
				Citalopram	Effective	rat		[100]
				Aspirin	Inactive	rat		[100]
		Carrageenan/kaolin (bilateral)	Vertical	-		rat	Only slight decrease in activity was observed	[109]
		CFA (bilateral)	Vertical	Ibuprofen	Effective	rat	More sensitive than reflexive outcomes	[109]
				Celecoxib	Effective	rat	More sensitive than reflexive outcomes	[109]
				Rofecoxib	Effective	rat	Similar to reflexive outcomes	[109]
				Piroxicam	Effective	rat		[109]
				Dexamethasone	Effective	rat		[109]
				Morphine (low dose)	Effective	rat	More sensitive than reflexive outcomes	[109]
				Morphine (high dose)	Inactive	rat		[109]
				Gabapentin	Inactive	rat		[109]
				Amitriptyline	Inactive	rat		[109]
				Amphetamine	Inactive	rat		[109]
	Osteoarthritis (early phase)	MIA (bilateral)	Vertical	Celecoxib	Effective	rat		[111]
				Naproxen	Effective	rat		[111]
	Osteoarthritis (late phase)	MIA (bilateral)	Vertical	Morphine (low dose)	Effective	rat		[111]
				Morphine (high dose)	Inactive	rat	Sensitive to side effects induced by morphine	[111]
				Tramadol	Effective	rat	Sensitive to side effects induced by tramadol	[111]
				Celecoxib	Inactive	rat		[111]
				Naproxen	Inactive	rat		[111]
				Acetaminophen	Inactive	rat		[111]
				Gabapentin	Inactive	rat		[111]
				Amitriptiline	Inactive	rat		[111]
	Postoperative pain	Laparotomy	Global	Morphine (i.t.)	Effective	rat	More sensitive than tactile allodynia	[125]
				Ketorolac (i.t.)	Effective	rat		[125]
				Morphine + ketorolac (i.t.)	Effective	rat		[125]
	Visceral	Acetic acid (i.p.)	Horizontal	Morphine	Effective	mouse	Less sensitive than writhing	[114]
				Caffeine	Effective	mouse	Due to locomotor (nonanalgesic) effects. Easy to control in noninjured mice.	[114]
				Haloperidol	Inactive	mouse		[114]
		Cyclophosphamide	Both	-		mouse		[16]
	Peripheral neuropathy	CCI	Vertical	Gabapentin	Effective	rat		[112]
				Duloxetine	Effective	rat		[112]
	Central pain	Contusion injury	Both	Gabapentin	Effective	rat		[113]
**Burrowing**	Inflammation	CFA	-	Ibuprofen	Effective	rat	More sensitive than heat hyperalgesia	[117]
	Postoperative pain	Laparotomy	-	Carprofen	Effective	mouse		[118]
	Peripheral neuropathy	TNT	-	Gabapentin (low dose)	Effective	rat		[117]
			-	Gabapentin (high dose)	Inactive	rat	Sensitive to the sedative (adverse) effect of gabapentin	[117]
		SNT	-	-		rat		[117]
		PSNL	-	-		rat		[117]
**Running wheels**	Inflammation	CFA (bilateral)	-	Naproxen	Effective	mouse	More sensitive than tactile allodynia	[52]
				Ibuprofen	Effective	Mouse		[52]
				Diclofenac	Effective	Mouse		[52]
				Celecoxib	Effective	Mouse		[52]
				Prednisolone	Effective	mouse		[52]
				Morphine (low dose)	Effective	Mouse		[52]
				Morphine (high dose)	Inactive	Mouse		[52]
				Caffeine	Inactive	mouse		[52]
	Osteoarthritis	MIA	-	-		rat	Only a percentage of animals were affected	[121]
	Visceral	Acetic acid (i.p.)	-	Morphine	Effective	mouse		[120]

The injuries were induced unilaterally unless otherwise indicated between brackets in the column headed “Injury”. The type of locomotion is indicated only for exploratory behavior, and was classified as horizontal, vertical, global (when horizontal and vertical were not distinguished) or both (when they were differentially analyzed to assess drug effects). Additional information about the drug treatment is included between brackets in the column headed “Drugs”. This information includes local administration (if applicable), and use of a low or high dose (if differential effects depending on the dose were seen). i.t.: Intrathecal administration.

**Table 5.** Summary of Studies Focused on Thermal Avoidance to Heat or Cold Stimuli

Avoidance to	Pathological State	Injury	Drug	Effect	Species	Notes	References
Heat	Acute	No injury	Morphine	Effective	rat	More sensitive than licking/ guarding in the hot plate	[130-132]
Cold	Cold-sensitizer	Icilin	-		rat		[136]
	Peripheral neuropathy	CCI (bilateral)	-	-	-		[137]
		CCI	Gabapentin	Effective	mouse		[129]
			Amitriptyline	Inactive	mouse	Amitriptyline altered locomotion at the dose tested	[129]
		PSL	-		mouse		[129]
		CCS	-		mouse		[129]
Heat (orofacial)	Inflammation	Carrageenan (bilateral)	Morphine		rat		[138]
	Heat-sensitizer	Capsaicin (bilateral)	Morphine		rat		[139]
Cold (orofacial)	Cold-sensitizer	Icilin (intra-cisterna magna)*	-		rat		[136]
	Cold-sensitizer	Menthol (bilateral)	-		rat		[140]

Experiments that tested thermal sensitivity in the face are identified as “orofacial” in the first column. Injuries were induced unilaterally unless otherwise indicated between brackets in the column headed “Injury”.

* The route of administration of icilin in studies of orofacial pain is indicated due to its peculiarity.

**Table 6.** Summary of Drug Effects on Place Preference to Pain or Analgesia

	Disease	Injury	Drugs/Treatments	Effects	Specie	Notes	References
**Conditioned place aversion by pain**	Chemical nociception (visceral)	Acetic acid	Timolol (amygdala or stria terminalis)	Effective	Rat	No effects on nociceptive behaviors	[144,145]
			Clonidine (amygdala)	Effective	Rat		[144,145]
	Chemical nociception (somatic)	Formalin	Timolol	Effective	Rat	No effects on nociceptive behaviors	[143-145]
	Inflammation	Carrageenan	Morphine	Effective	Rat	More sensitive than mechanical hyperalgesia	[146]
			Oxycodone	Effective	Rat		[147]
			Tramadol	Effective	Rat		[147]
			Ibuprofen	Effective	Rat		[147]
			Pregabalin	Effective	Rat		[147]
**Conditioned place aversion by pain (mechanical stimulation)**	Inflammation	Carrageenan	Aspirin	Effective	Rat	More sensitive than tactile allodynia	[149]
			Morphine	Effective	Rat		[152]
		CFA	Celecoxib	Effective	Rat	More sensitive than tactile allodynia	[153]
			Duloxetine	Effective	Rat		[153]
			Diclofenac	Effective	Rat		[153]
			Scopolamine	Inactive	Rat		[153]
			Fluoxetine	Inactive	Rat	Motor impairment effects	[153]
	Peripheral neuropathy	SNL	Morphine (low dose)	Effective	Rat		[151,152,165]
			Morphine (high dose)	Inactive	Rat	Probably because of alterations in the motility	[165]
			Morphine (anterior cingulate cortex)	Effective	Rat	No effects on mechanical allodynia	[151]
			Gabapentin	Effective	Rat	Similar sensitivity to mechanical allodynia	[165]
		CCI	Morphine	Effective	Rat	More sensitive than mechanical allodynia or hyperalgesia	[154]
			Gabapentin	Effective	Rat		[154]
			8-OH-DPAT (5HT_1A_ agonist)	Effective	Rat		[154]
			Duloxetine	Effective	Rat	No effects on mechanical allodynia or hyperalgesia	[154]
			Gaboxadol	Inactive	Rat	Also inactive in mechanical allodynia or hyperalgesia	[154]
			WIN55,212-2 (CB agonist)	Inactive	Rat		[154]
**Conditioned place preference by analgesia**	Inflammation	CFA	Morphine	Effective	Rat		[157]
			Indomethacin	Inactive	Rat	Also inactive in the hot-plate test	[157]
			MK-801 (NMDA antagonist)	Effective	Rat		[157]
			Des-Arg9,(Leu8)-B (bradykinin B1 antagonist)	Effective	Rat		[158]
			HOE 140 (bradykinin B2 antagonist)	Inactive	Rat		[158]
			Lidocaine (intraarticular)	Effective	Rat		[160]
			Clonidine (i.t.)	Effective	Rat		[160]
			AMG9810 (TRPV1 antagonist)	Inactive	Rat		[160]
	Peripheral neuropathy	SNL	Clonidine (i.t.)	Effective	Rat		[159]
			Lidocaine (RVM)	Effective	Rat		[159]
			Adenosine (i.t.)	Inactive	Rat		[159]
		SNI	Clonidine (i.t.)	Effective	Rat		[162]
	Central pain	Electrolytic lesion in the spinal cord	Clonidine (i.t.)	Effective	Rat		[161]
			Motor cortex manipulation	Effective	Rat		[161]
	Osteoarthritis (late stage)	MIA	Lidocaine (intraarticular)	Effective	Rat		[61]
			AMG9810 (TRPV1 antagonist)	Inactive	Rat		[61]
			HC030031 (TRPA1 antagonist)	Inactive	Rat		[61]
			Clonidine (i.t.)	Effective	Rat		[85]

Additional information about drug treatment is included between brackets in the column headed “Drugs”. This information includes local administration (if applicable), and use at a low or high dose (if differential effects depending on the dose were seen). i.t.: intrathecal administration. RVM: rostral ventromedial medulla

**Table 7.** Summary of Drug Effects in Self-administration Experiments Under a Painful Condition

Disease	Injury	Drugs	Intake/Rate of Responding	Route of Administration	Species	References
Inflammatory	CFA	Fentanyl	Increased	Drinking water	rat	[167-169]
		Suprofen	Increased	Drinking water	rat	[167]
Postoperative pain	Cecal manipulation	Ibuprofen	Increased	Drinking water	mouse	[170]
Peripheral neuropathy	PSNL	Fentanyl	Unaltered	Drinking water	rat	[168]
		S1RA (sigma-1 antagonist)	Increased	Intravenous	mouse	[174]
	SNL	Clonidine	Increased	Intrathecal	rat	[172]
		Morphine	Maintained	Intravenous	rat	[175]
		Morphine (low dose)	Decreased	Intravenous	rat	[175]
		Fentanyl	Maintained	Intravenous	rat	[175]
		Fentanyl (low dose)	Decreased	Intravenous	rat	[175]
		Hydromorphone	Maintained	Intravenous	rat	[175]
		Hydromorphone (low dose)	Decreased	Intravenous	rat	[175]
		Methadone	Maintained	Intravenous	rat	[175]
		Methadone (low dose)	Decreased	Intravenous	rat	[175]
		Heroin	Maintained	Intravenous	rat	[175]
		Heroin (low dose)	Decreased	Intravenous	rat	[175]
	SNI	(R,S)-AM1241 (CB2 agonist)	Increased	Intravenous	rat	[173]

Additional information about drug treatment is included between brackets in the column headed “Drugs”. This information includes use of a low or high dose (if differential effects depending on the dose were seen) as well as the target of nocommercially available drugs identified by a code number.

**Table 8.** Summary of Studies Focused on the Effects of Pain in Anxiety-like Behaviors

Disease	Injury	Behavioral Test	Altered by Injury	Drugs	Effect	Specie	Notes	References
Inflammation	CFA	Elevated plus maze	yes	Etizolam	Effective	mouse	Did not alter mechanical allodynia or thermal hyperalgesia	[211]
			yes	Morphine	Effective	rat		[206]
			yes	Diazepam	Effective	rat		[206]
		Elevated zero maze	no			mouse		[101]
		Light-dark	no			rat		[206]
			yes	Etizolam		mouse	Did not alter mechanical allodynia or thermal hyperalgesia	[211]
		Marble-burying behavior	no			mouse		[101]
		Open field	no			mouse		[101]
			yes			rat		[212]
			yes			rat		[206]
		Social interaction	no			mouse		[101]
			yes			rat		[206]
	Kaolin/ Carrageenan	Elevated plus maze	yes	NBI27914 (CRF1 antagonist)	Effective	rat		[208]
			yes	NBI27914 (CRF1 antagonist, amygdala)	Effective	rat		[208]
Peripheral neuropathy	CCI	Elevated plus maze	no			rat		[112]
			yes	Morphine	Effective	rat		[204]
			yes	Gabapentin	Effective	rat		[204]
			yes	Midazolam	Effective	rat	Did not alter mechanical allodynia	[204]
		Elevated zero maze	no			mouse		[101]
		Marble-burying behavior	no			mouse		[101]
		Open field	no			mouse		[101]
			yes	Duloxetine	Effective	rat		[112]
			yes	Gabapentin	Effective	rat		[112]
		Social interaction test	no			rat		[112]
	PSNL	Elevated plus maze	no			rat		[204]
			yes	Imipramine	Effective	mouse	More sensitive than mechanical allodynia	[205]
			yes	Milnacipram	Effective	mouse		[205]
			yes	Paroxetine	Effective	mouse		[205]
			yes	Paroxetine (amygdala or cingulate cortex)	Effective	rat		[205]
			yes	Etizolam	Effective	mouse		[211]
			yes*			mouse		[202]
		Light-dark	yes	Imipramine	Effective	mouse	More sensitive than mechanical allodynia	[205]
			yes	Milnacipram	Effective	mouse		[205]
			yes	Paroxetine	Effective	mouse		[205]
			yes			mouse		[211]
		Open field	no			mouse		[202]
			yes			rat		[203]
	Sciatic nerve cuffing	Elevated plus maze	yes			mouse		[213]
		Light-dark	yes			mouse		[213]
		Marble-burying behavior	yes			mouse		[213]
		Novelty suppressed feeding	yes			mouse		[213]
	SNI	Elevated plus maze	no			rat		[214]
			yes			rat		[215]
			yes			rat		[162]
			yes			rat		[216]
		Elevated zero maze	no			mouse		[101]
		Marble-burying behavior	no			mouse		[101]
		Open field	no			rat		[214]
			no			mouse		[217]
			no			mouse		[218]
			no			mouse		[101]
			yes	Gabapentin	Effective	rat		[203]
			yes			rat		[215]
		Social interaction	no			mouse		[101]
	SNL	Elevated plus maze	no			rat		[219]
			yes			mouse		[220]
		Light-dark	no			rat		[219]
			yes			mouse		[220]
		Open field	no			rat		[219]
			yes			mouse		[220]
	*Varicella zoster* virus	Open field	yes	Gabapentin	Inactive	rat		[203]
	Zalcitabine (antiretroviral)	Open field	yes	Morphine	Effective	rat		[221]
			yes	Gabapentin	Effective	rat		[221]
			yes	Diazepam	Effective	rat		[221]
Postoperative pain	Plantar incision	Elevated plus maze	yes			rat		[222]
			yes	Morphine	Effective	rat		[207]
			yes	Gabapentin	Effective	rat		[207]
		Open field	yes			rat		[207]
			yes			rat		[222]
Visceral	Acetic acid	Elevated plus maze	yes			mouse		[223]
		Open field	yes			mouse		[223]

Additional information about drug treatment is included between brackets in the column headed “Drugs”. This information includes local administration (if applicable) and the target of noncommercially available drugs identified by a code number.

* The injury unexpectedly induced the opposite effect (anxiolysis).

**Table 9.** Summary of Studies Focused on the Effects of Pain in Depressive-like Behaviors

Disease	Injury	Type of Test	Behavioral Test	Altered by Injury	Drugs	Effective	Specie	Notes	Reference
Inflammation	CFA	Despair	Forced swimming	no			mouse		[101]
			Forced swimming	yes			rat		[212]
		Reward	Sucrose preference	no			mouse		[101]
Peripheral neuropathy	CCI	Despair	Forced swimming	no			mouse		[101]
			Forced swimming	yes	Desipramine	yes	rat	Little effect on mechanical allodynia	[238]
			Forced swimming	yes	GW405833 (CB2-selective agonist)	yes	rat	Similar effect on mechanical allodynia	[238]
		Reward	Saccharin preference	no			rat		[112]
	PSNL	Despair	Tail suspension	no			mouse		[202]
	Sciatic nerve cuffing	Despair	Forced swimming	yes			mouse		[213]
		Self care	Splash	yes			mouse		[213]
	SNI	Despair	Forced swimming	yes			rat		[214]
			Forced swimming	yes	Ketamine	yes	rat		[237]
			Forced swimming	yes			rat		[215]
			Forced swimming	yes	Oxytocin (i.c.v.)	yes	mouse	No effect on mechanical allodynia	[218]
			Forced swimming	yes	Metyrapone (glucocorticoid synthesis inhibitor)	yes	mouse		[217]
			Forced swimming	yes	IL-1ra (IL-1 receptor antagonist, i.c.v.)	yes	mouse		[217]
		Reward	Sucrose preference	no			mouse		[101]
			Sucrose preference	yes	Ketamine	yes	rat	No effect on mechanical or cold allodynia	[237]
	SNL	Despair	Forced swimming	no			rat		[219]
			Forced swimming	yes			mouse		[220]
Visceral	Acetic acid	Reward	Saccharin preference	yes	Ibuprofen	yes	mouse		[115]
			Saccharin preference	yes	Caffeine	no	mouse		[115]

Additional information about drug treatment is included between brackets in the column headed “Drugs”. This information includes local administration (if applicable) and the target of noncommercially available drugs identified by a code number. i.c.v.: Intracerebroventricular administration

**Table 10.** Summary of Studies Focused on the Impact of Pain in Sleep

Disease	Injury	Behavior Altered by Injury	Drug	Effect	Specie	Notes	References
Inflammatory	CFA	Yes			rat		[239,240,242]
		Yes	Acetylsalicylic acid	Detrimental	rat	Reduced non-REM sleep in normal rats	[241]
		Yes	Acetaminophen	Effective	rat	Reduced non-REM sleep in normal rats	[241]
Inflammatory (orofacial)	CFA	Yes	Indomethacin	Effective	rat	No effects in normal rats	[243]
		Yes	L-NAME (nitric oxide synthase inhibitor)	Effective	rat	Increased REM sleep in normal rats	[244]
		Yes	Etoricoxib	Effective	rat	Decreased sleep latency in normal rats	[245]
Inflammatory	Uric acid	Yes			rat		[251]
Osteoarthritis	MIA	Yes			rat	Most changes between days 10 and 28 (late phase)	[246]
Peripheral neuropathy	CCI	No			rat		[247]
		Yes			rat	Apparent changes mostly in the first week	[248]
		Low frequency			rat	Only a subpopulation showed alterations	[249]
	PSNL	Yes	SNAP-5114 (GAT-3 inhibitor) (intracingulate cortex)	Effective	mouse		[250]

Experiments that tested sleep alterations after inflammation in the temporomandibular joint are indicated as “orofacial” in the first column. Additional information about the drug treatment is included between brackets in the column headed “Drugs”. This information includes local administration and the target of noncommercially available drugs (if applicable).

**Table 11.** Summary of Nonreflexive Outcomes used for Preclinical Pain Testing in Tonic and Pathological Pain Models

Pain State Modeled	Experimental Manipulation	Reflexive Outcome	Nonreflexive Outcome
Tonic pain	Formalin	Licking/flinching of the paw	Conditioned place aversion
	Acetic acid (i.p.)	Stretching of the body	Decreased exploratory activity
			Decreased wheel running
			Conditioned place aversion
			Anhedonia
Postoperative pain	Plantar incision	Thermal and mechanical hypersensitivity	Weight bearing asymmetry (if performed in the paw)
	Other surgical procedures	-	Decreased exploratory activity
			Increased analgesic self-administration
Inflammatory pain	Injection of inflammatory compound	Thermal and mechanical hypersensitivity	Weight bearing asymmetry
			Gait alterations
			Grip strength deficits
			Decreased exploratory activity
			Decreased burrowing behavior
			Decreased wheel running
			Decreased home cage activity
			Heat avoidance
			Analgesic self-administration
			Conditioned place aversion by pain
			Conditioned place preference by analgesia
			Increased anxiety and depression related behaviors?
			Sleep alterations
Osteoarthritis	MIA	Thermal and mechanical hypersensitivity	Weight bearing asymmetry
			Grip strength deficits
			Decreased home cage activity
			Decreased exploratory activity
			Conditioned place preference by analgesia
Cancer pain		Thermal and mechanical hypersensitivity	Grip strength deficits
Peripheral neuropathy	Traumatic peripheral nerve injury	Thermal and mechanical hypersensitivity	Decreased burrowing behavior
			Cold avoidance
			Analgesic self-administration
			Conditioned place aversion by pain
			Conditioned place preference by analgesia
			Increased anxiety and depression related behaviors?
			Sleep alterations?
Central neuropathy	Central nervous system damage	Mechanical hypersensitivity	Decreased exploratory activity
			Conditioned place preference by analgesia

?: Unclear at the moment i.p.: Intraperitoneal administration See Tables 1-10 for details and references.
